# Carotid atherosclerosis and its relationship to coronary heart disease and stroke risk in patients with type 2 diabetes mellitus

**DOI:** 10.1097/MD.0000000000008151

**Published:** 2017-09-29

**Authors:** Yan Wu, Jie He, Xue Sun, Yi-Ming Zhao, Han-Yu Lou, Xiao-li Ji, Xiao-Hong Pang, Li-Zhen Shan, Ying-Xiu Kang, Jun Xu, Song-Zhao Zhang, Yong-Jian Wang, Yue-Zhong Ren, Peng-Fei Shan

**Affiliations:** a Department of Endocrinology and Metabolism, the Second Affiliated Hospital ZheJiang University College of Medicine; b Department of Endocrinology and Metabolism, Zhejiang Greentown Cardiovascular Hospital; c Department of Ultrasound; d Department of Clinical Laboratory, the Second Affiliated Hospital ZheJiang University College of Medicine, Hangzhou, Zhejiang, P.R. China.

**Keywords:** carotid atherosclerosis, coronary heart disease, diabetes mellitus, stroke

## Abstract

Carotid atherosclerosis (CA) and carotid plaque (CP) are highly correlated with cardiovascular disease. We aimed to determine the prevalence of CA and CP and their relationship with 10-year risks of stroke and coronary heart disease (CHD) in type 2 diabetes mellitus (T2DM).

We studied 1584 T2DM patients aged 20 years and older. CA and CP were detected using ultrasonography. Ten-year stroke and CHD risk were determined using the United Kingdom Prospective Diabetes Study (UKPDS) risk engine.

The prevalence of CA and CP increased gradually with age. Men had a higher prevalence of CA than women (CA: 58.18% vs 51.54%, *P* < .01). The 10-year CHD risk (27.9% vs 15.4%, *P* < .001) and stroke risk (15.2% vs 5.70%, *P* < .001) were higher in patients with CA than that of those without CA. Compared with patients without CA, the odds ratios (ORs) of CHD in CA and CP group were 4.47 and 10.78 for men, and 4.19 and 5.20 for women, respectively; in the case of stroke, the OR in CA and CP group were 8.83 and 12.07 for men, and 4.35 and 4.90 for women, respectively (*P* < .001 for all). Multivariate binary logistic regression analysis showed that CA was an independent risk factor for CHD [OR = 2.66, 95% confidence interval (95% CI), 2.05–3.46, *P* < .001] and stroke (OR = 3.11, 95% CI, 2.38–4.07, *P* < .001).

CA and CP were prevalent in patients with T2DM and positively correlated with 10-year CHD and stroke risk. CA was an independent risk factor for 10-year CHD risk.

## 1 Introduction

Diabetes mellitus is now an important public health concern. Worldwide, the number of individuals living with diabetes mellitus is likely to be as high as 592 million by 2035 according to the International Diabetes Federation.^[1]^ The prevalence of diabetes is also up to 9.7% in China.^[2]^ There is consensus that diabetes is the coronary artery disease equivalent and is one of the principal cardiovascular disease (CVD) risk factors. The risk of CVD is 2 to 4 times higher in patients with type 2 diabetes mellitus (T2DM) than in the general population,^[3]^ and in recent years, CVD has become the leading cause of death in these patients in recent years.^[4]^

Interestingly, CVD does not develop in all patients with T2DM. T2DM patients have many CVD risk factors in addition to hyperglycemia. Among T2DM patients with CVD, 77% to 87% have hypertension, 74% to 81% have increased low-density lipoprotein cholesterol (LDL-c) levels, and 62% to 67% are overweight.^[5]^ This high prevalence of other CVD risk factors is correlated with the increased CVD risk in patients with T2DM. Studies have indicated that hypertension has an important influence on cardiovascular outcome in T2DM patients. The United Kingdom Prospective Diabetes Study (UKPDS) showed in a multivariate analysis that the strongest independent risk factor for CVD was elevated LDL-c, followed by reduced high-density lipoprotein cholesterol (HDL-c) level.^[6]^ In addition, cigarette smoking is an important risk factor for CVD in these patients.^[7]^ Up to now, it is still challenging to identify individual T2DM patients who are at a high risk for CVD.

Diabetes also has a close relation to subclinical atherosclerosis.^[8]^ Carotid atherosclerosis (CA) detected ultrasonically occurs in arteries with intimal hyperplasia. Carotid intima–media thickness (CIMT), which can be used to predict the risk of developing coronary artery stenosis in patients who are asymptomatic, is significantly greater in those with T2DM than in the general population.^[9]^ Measurement of CIMT is simple, noninvasive and a valuable means of identifying high-risk individuals both with and without diabetes.^[10]^ The presence of carotid plaque (CP) is an indicator of advanced atherosclerosis and can be used to predict cardiovascular risk,^[11]^ being associated with major adverse cardiovascular events in patients with T2DM.^[12]^ Studies also suggest that low gray-scale median of plaque echogenicity and low eicosapentaenoic acid/arachidonic acid ratio might be useful for predicting the risk of CVD in T2DM patients.^[13]^

We have previously investigated CA and CP in the general population.^[14]^ There has been little research on CA and CP in patients with T2DM in China, and most studies have been conducted in populations in clinical settings and do not describe age- or gender-related differences in CA and CP.^[15,16]^ Li et al^[15]^ explored the prevalence and clinical characteristics of CA in newly diagnosed patients. Another study investigated the prevalence of CA and CP in patients with both T2DM and hypertension.^[16]^ Furthermore, there is a lack of data on the use of CA to predict CHD and stroke risk in China. Thus, we aimed to assess the age- and gender-specific prevalence of CA and CP, and their correlation with 10-year risks of stroke and CHD estimated by UKPDS risk engine^[17,18]^ in patients with T2DM.

## 2 Methods

### 2.1 Subjects

One thousand, five hundred eighty-four T2DM patients of age 20 years and above were diagnosed according to the World Health Organization 2006 criteria, that is, fasting plasma glucose ≥126 mg/dL and/or 2 hours plasma glucose after 75 g oral glucose tolerance test ≥200 mg/dL^[19]^; or the use of any antidiabetic drug, including insulin and oral hypoglycemic agents. Patients with CHD, stroke, and overt hyperthyroidism and hypothyroidism were excluded from this study. Patients were admitted to the Second Affiliated Hospital of Zhejiang University College of Medicine, Zhejiang, China, from August 2008 to April 2013. The study was approved by the Ethics Committee of the Second Affiliated Hospital of Zhejiang University College of Medicine. Informed consent was obtained before examination.

All participants underwent clinical assessment including medical history and physical examination. For weight measurement, they were instructed to wear lightweight clothes. Body mass index (BMI) was calculated as body weight in kilograms divided by height in meters squared. A plane around the abdomen at the level of the halfway point between the lowest edge of the ribs and the iliac crest was used to determine abdominal circumference. Blood pressure was measured twice for each participant using a Kenz BPM SP-1 automatic blood pressure device (Suzuken Co., Ltd, Nagoya, Japan). Measurements were made on the right arm after the participant rested for ≥5 minutes; the mean value was used in the report.

### 2.2 Biochemical tests

Samples of venous blood were obtained between 6:00 and 8:00 am after overnight fasting. Triglycerides, total cholesterol, fasting plasma glucose, LDL-c, and HDL-c levels were determined with an AU4500 automatic chemistry analyzer (Olympus Corporation, Tokyo, Japan). Glycated hemoglobin (HbA1c) was measured with a TOSOH HLC-723G8 automatic glycohemoglobin analyzer (Tosoh Corporation, Yamaguchi, Japan) and fasting C-peptide with an ADVIA Centaur XP immunoassay System (Siemens Inc., Munich, Germany).

### 2.3 Assessment of CA

The carotid artery was assessed by 6 experienced sonographers using a B-mode ultrasound imaging unit (Alpha10; Aloka Co., Ltd, Tokyo, Japan) with a 10 MHz linear arrayprobe. The sonographers, certified by the Ministry of Health of China, did not know the clinical status or biochemical test results of the participants. Longitudinal 2-dimensional images were obtained and used to determine CIMT at the far wall of the common carotid artery on 2 sides over 10 mm proximal to the bifurcation.^[20]^

CIMT was measured at the end of diastole as the distance between the leading edges of the first and second echogenic lines. These lines represent the lumen–intima interface and the collagen-containing upper layer of the tunic adventitia, respectively. For analysis, we used the maximum value measured between the 2 sides of the common carotid artery. The CIMT measurements were repeated 3 times a day to assess in vivo precision and reexamined after 2 weeks in 16 subjects. The IMT measurements’ mean intra- and interobserver coefficients of variation were 5.9% and 7.5%, respectively. CA was considered to be present when the CIMT was greater than 1.0 mm.^[21]^ CP was defined as CIMT greater than 1.5 mm at the carotid bulbor at the internal or common carotid artery with or without the presence of flow disturbance.^[22]^

### 2.4 Calculation of risk using the UKPDS risk engine

Ten-year CHD and stroke risks were calculated for each patient using the UKPDS risk engine (v. 2.0). The model incorporates risk factors, including sex, age, HbA1c level, race, HDL-c, current smoking status, total cholesterol, systolic blood pressure (SBP), and atrial fibrillation status.^[17,18]^ According to the results, participants in the study were considered to be at low (≤10%), intermediate (10–20%), or high (>20%) risk of CHD.

### 2.5 Statistical analysis

Data were analyzed using SPSS 17.0 (IBM, Armonk, NY). The prevalence of CA and CP was determined among all participants, in males and females, and according to 10-year and 20-year age groups. Continuous variables were presented as the mean ± standard deviation (SD) or the mean with 95% confidence interval (95% CI); categorical variables were presented as the frequency with the percentage given in parentheses. Mann–Whitney tests or independent *t* tests were used to compare continuous variables among groups and χ^2^ tests were used to compare proportional data. Categorical parameters and risk estimation were evaluated by Chi-squared tests. Correlations between categorical variables and risk factors were analyzed by binary logistic regression analysis. All statistical tests were 2-tailed and *P* < .05 was considered significant.

## 3 Results

### 3.1 Characteristics of T2DM patients with and without CA

Table 1 summarizes biochemical and anthropometric data for the participants according to sex and the presence of CA. Patients with CA were older, had a significantly longer duration of diabetes and higher SBP, and included more ex-smokers than patients without CA. Among men with CA, waist circumference, serum total cholesterol, LDL-c, and HDL-c were similar to men without CA, triglycerides were lower, and there were more current smokers. Women with CA had significantly higher serum total cholesterol, LDL-c, and HDL-c levels and included more current smokers than those without CA. BMI, diastolic blood pressure, waist circumference, HbA1c, and fasting plasma glucose were similar in both sexes with or without CA. The prevalence of female smoker was quite low compared with male in our result (1.34% vs 40.3%, *P* < .001), which was similar to the epidemiological survey results in Zhejiang province in 2013, 0.78% for women, 50.78% for men.^[23]^ Multivariate binary logistic regression analysis showed that age [odds ratio (OR) = 1.086, 95% CI = 1.074–1.099; *P* = .000], SBP (OR = 1.007, 95% CI = 1.001–1.013; *P* = .019), duration of diabetes (OR = 1.025, 95% CI = 1.005–1.044; *P* = .012), female sex (OR = 0.467, 95% CI = 0.369–0.591; *P* = .000), and total cholesterol (OR = 1.150, 95% CI = 1.047–1.279; *P* = .005) were independently associated with CA.

**Table 1:**
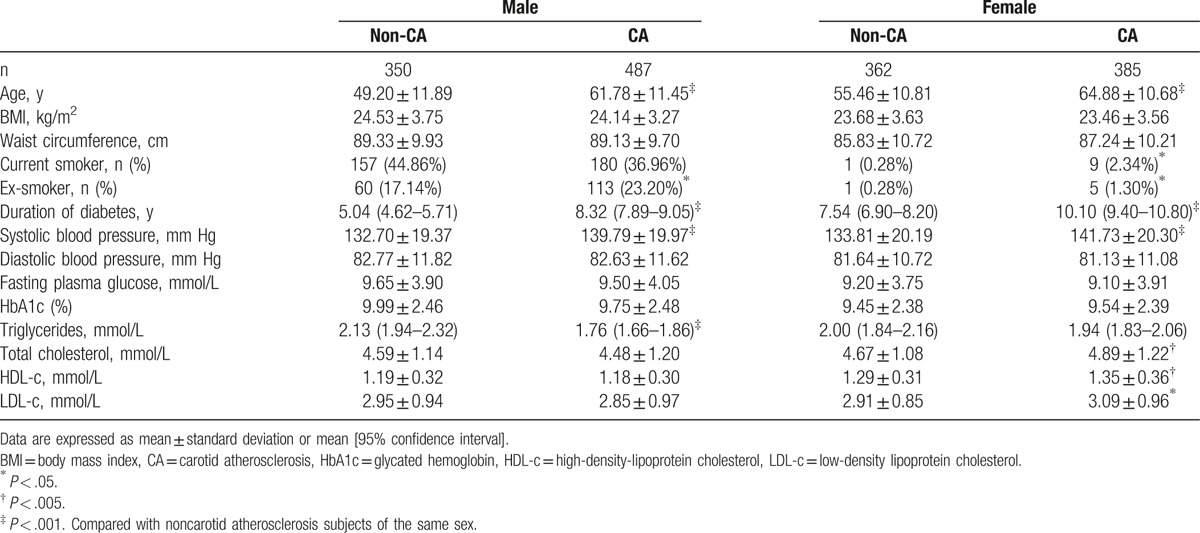
Anthropometric and biochemical data according to presence of CA and sex.

### 3.2 Prevalence of CA and CP

**Figure 1:**
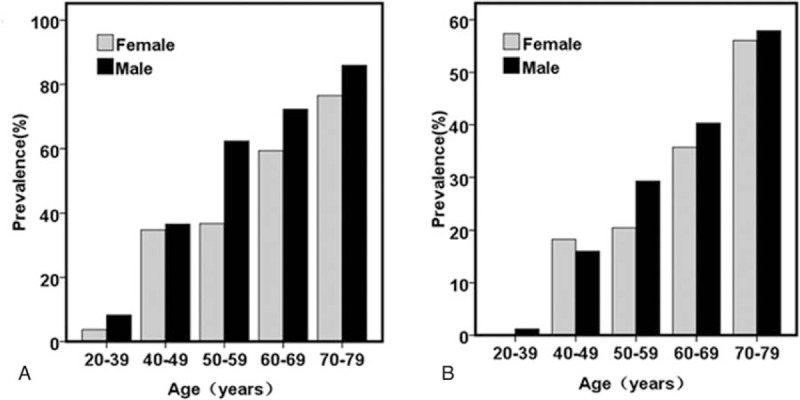
Age-related prevalence of carotid atherosclerosis (A) and carotid plaque (B) in male and female participants in the study.

Among all patients with T2DM, the prevalence of CA was higher in males than in females, whereas CP was similar between the 2 sexes (CA: 58.2% vs 51.5%, *P* < .01; CP: 31.4% vs 31.9%, *P* = NS). CA onset occurred during the 20 seconds in men; the prevalence of CA increased gradually from 8.24% at 20 to 39 years of age to 86.0% in individuals aged 70 to 79 years (Fig. 1A, B). The prevalence of CP was 1.18% at 20 to 39 years and 57.9% at 70 to 79 years. In women, the onset of CA occurred in the same decade as in men; its prevalence was 3.70% at 20 to 39 years and increased gradually to 76.5% at 79 to 79 years. CP occurred later in women than in men; the prevalence was 18.3% at 40 to 49 years and 56.1% at 70 to 79 years. The prevalence of CA was 1.79% to 25.6% higher in men than in women in the same age group over 50 years of age and that of CP was 1.83% to 8.82% higher.

According to our previous study,^[14]^ the prevalence of CA in T2DM is about 10% to 20% higher than that in general population and that of CP is 6.0% to 15.8% higher.

### 3.3 Relationship between CHD risk, stroke risk, and CA

Considering all participants, the average 10-year CHD and stroke risks were higher in patients with CA than that of those without CA (27.9% vs 15.4%for CHD risk, *P* < .001; 15.2% vs 5.70% for stroke risk, *P* < .001). CHD and stroke risk are displayed according to sex and the presence of CA in Table 2. In both sexes, individuals with CA had significantly higher CHD risk, fatal CHD risk, stroke risk, and fatal stroke risk than those without CA (*P* < .001 for all).

**Table 2:**
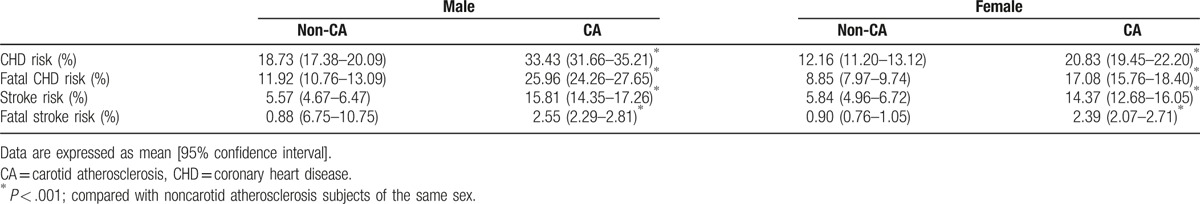
UKPDS score according to sex and presence of CA.

**Figure 2:**
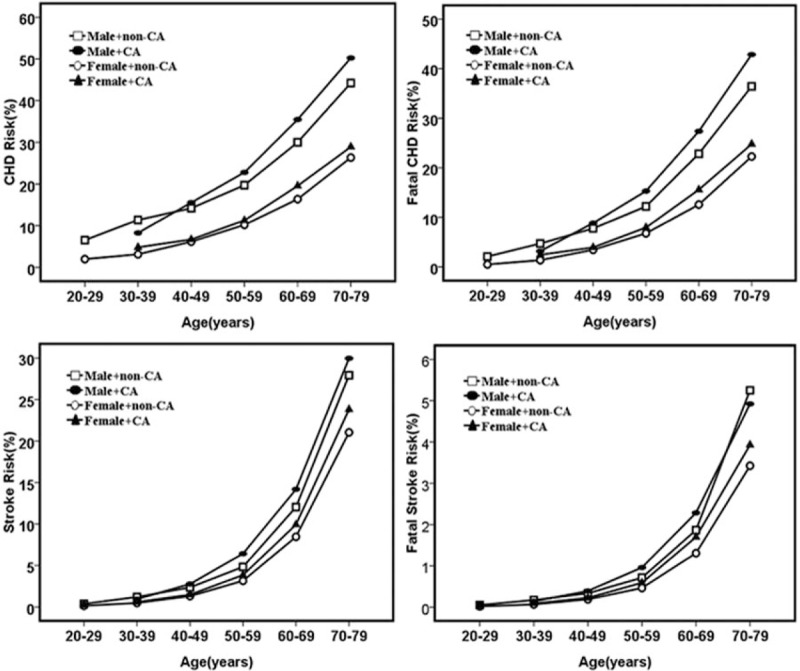
Age-related coronary heart disease (CHD), fatal CHD, stroke, and fatal stroke risks in male and female participants with carotid atherosclerosis (CA) and without carotid atherosclerosis (non-CA).

CHD risk and stroke risk increased with age. In the same decade of age after 40 years, patients with CA had higher CHD risk than those without CA of the same sex. A similar relationship existed for patients younger than 70 years with respect to stroke risk (Fig. 2). The impact of CA and CP on cardiovascular risk is summarized in Table 3. In both sexes, individuals with CA had a higher prevalence of high CHD and stroke risk than those without CA (*P* < .001 for all).

**Table 3:**
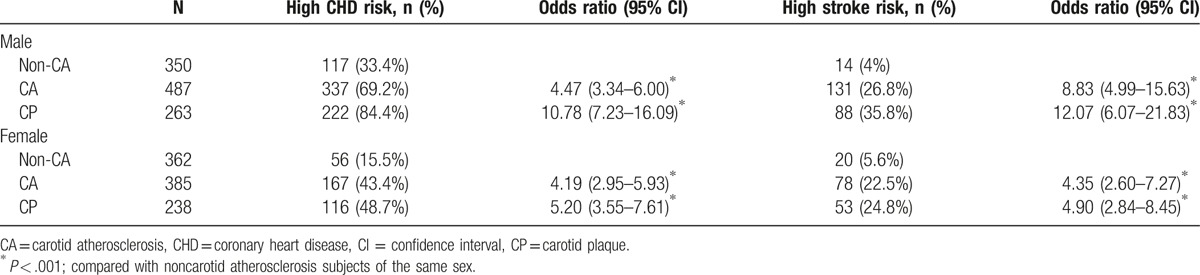
Impact of high CHD and stroke risk on CA and CP outcomes according to sex.

The prevalence and ORs of CHD and stroke increased with increasing CA in both sexes. Compared with patients without CA, the OR of CHD in CA and CP group were 4.47 (95% CI, 3.34–6.0) and 10.78 (95% CI, 7.23–16.09) for men, and 4.19 (95% CI, 2.95–5.93) and 5.20 (95% CI, 3.55–7.61) for women, respectively (*P* < .001 for all). In the case of stroke, the OR in CA and CP group were 8.83 (95% CI, 4.99–15.63) and 12.07 (95% CI, 6.07–21.83) for men, and 4.35 (95% CI, 2.60–7.27) and 4.90 (95% CI, 2.84–8.45) for women, respectively (*P* < .001 for all). We then performed a binary logistic regression analysis with the following dependent variables: age ≥50 years, CA, male sex (female 0, male 1), elevated total cholesterol (≥4.5 mmol/L), hypertension, reduced blood HDL-c levels [<1.04 mmol/L (men) or <1.29 mmol/L (women)]. The independent variables were as follows: UKPDS CHD risk (>20%, high risk, 1; ≤20%, 0) and stroke risk (>10%, high risk, 1; ≤10%, 0). The results showed that CA was an independent risk factor for CHD (OR = 2.66, 95% CI, 2.05–3.46, *P* = .000) and stroke (OR = 3.11, 95% CI, 2.38–4.07, *P* < .001), Table 4.

**Table 4:**
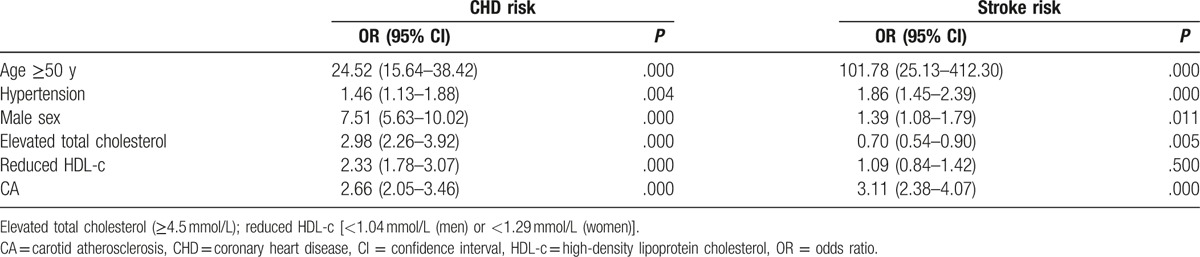
Multivariate binary logistic regression analysis of risk factors for CHD and stroke as estimated using the UKPDS risk engine.

In female patients, the average 10-year CHD and stroke risks of postmenopausal women were higher than pre-menopausal women [0.191 (0.181–0.202) vs 0.069 (0.060–0.078) for CHD risk, *P* < .001; 0.120 (0.111–0.136) vs 0.018 (0.013–0.23) for stroke risk, *P* < .001].

## 4 Discussion

In our sample of T2DM patients, we found that the incidence of both CP and CA increased with age and the incidences of CA and CP were higher in men than in women. Mean age, SBP, and duration of diabetes, which are risk factors for CHD, were higher in patients with CA than in those without CA. The estimated 10-year risk of CHD and stroke risk increased with age. Among older patients, those with CA had a higher CHD risk than those without CA. We also found that CA was an independent risk factor for CHD risk after adjusting for age, SBP, total cholesterol, and HDL-c.

In this study, the prevalence of CA was 55.1%, and in every decade, the incidence was higher than that in the general population as determined in our previous research.^[14]^Several studies have demonstrated that patients with T2DM are at a higher risk for atherosclerosis than healthy subjects.^[24]^ There were also other studies on the prevalence of CA in patients with T2DM of different clinical setting. We found that the presence of CA was independently associated with age, duration of diabetes, SBP, male sex, and total cholesterol in patients with T2DM. In a research in which the percentage of male patients was lower than our study, the prevalence of CA detected by ultrasound examination in hospitalized T2DM patents was 44.4%.^[25]^ This value is lower than ours. This also supports that male sex is a risk factor for CA.^[26]^ In another study in newly diagnosed T2DM patients, the prevalence of CA was 35.8%, which is lower than our finding.^[15]^ This indicates that the prevalence of CA may be associated with the duration of diabetes, as has been demonstrated in our research. In older T2DM patients with hypertension, researchers found that CA was quite common, being observed in 62.1% of subjects, which is more than in our study.^[16]^ Hypertension and age were risk factors for CA in our T2DM patients, which is consistent with other studies.^[27]^

In the general population, studies have shown that CIMT was closely related to cardiovascular risk and stroke risk. Researchers found an approximately 40% increase in CHD risk with each SD increase in CIMT.^[28,29]^ CIMT is an even more powerful predictor for stroke risk, with an approximate increase in cerebrovascular disease of 50% to 80% for each SD increase.^[30]^ CIMT was measured for every participant with an average follow-up time of 5.2 years in the Atherosclerosis Risk in Communities (ARIC) study. CHD was 1.85 times more prevalent in men and 5.07 times more prevalent in women with CA than in patients without CA.^[31]^ In a previous ARIC study that examined the relationship between CIMT and cerebrovascular disease over a mean of 7.2 years, ischemic cerebrovascular disease was more prevalent in patients with CA than in those without (2.02 times greater in women and 1.78 times greater in men).^[32]^

T2DM patients are at a greater risk of developing CVD than the general population and T2DM leads to increased morbidity and mortality from CVD. Epidemiologic data suggest that the cardiovascular risk attributable to T2DM remains about 2-fold increased even after adjustment for traditional risk factors such as hyperglycemia, hypertension, and hyperlipidemia.^[33]^ Therefore, it is crucial to identify patients at a high residual risk to enable the intensification of preventive therapies in these individuals. The greater risk for CVD in T2DM patients cannot entirely be explained by traditional risk factors alone, and there have been efforts to identify and understand the link between diabetes and “non-traditional” CVD risk factors such as endothelial dysfunction, inflammation, impaired fibrinolysis, increased homocysteine levels, microalbuminuria, and vascular wall abnormalities. The relationship between CA and CHD risk has also been studied in T2DM patients in recent years.

Another important result in our study was that after adjustment for age and other risk factors for CHD, CA was independently correlated with CHD risk and stroke risk in our study. In the study by Seon et al, ^[27]^ 10-year CHD and stroke risks calculated using the UKPDS risk engine were positively associated with CIMT, which is similar to our results. 10-year CHD risk and 10-year stroke risk both increased with age.

Our study also has limitations. First, the UKPDS risk engine was developed using data obtained from newly diagnosed T2DM patients, whereas some participants in our study had diabetes of longer duration; the UKPDS risk engine is highly sensitive for predicting CHD risk in Chinese patients with diabetes, with convergent validity and good feasibility,^[34]^ thus, using it to predict CHD and stroke risk in the participants in our study is reliable. Second, our participants were all inpatients whose glycemic control was unsatisfactory. Worldwide, most patients with T2DM fail to achieve adequate glycemic control,^[25]^ so to some extent, our data could represent the general T2DM population. Third, this is a cross-sectional study, and a further prospective study is required to investigate the development and progression of CA and its relationship to CVD risk in T2DM patients.

In summary, we demonstrate that the prevalence of CA in T2DM patients was higher in men, was higher than in the general population, and increased with age. CA and CP were correlated with 10-year CHD and stroke risk according to the UKPDS risk engine in patients with T2DM. Furthermore, CA was independently associated with 10-year CHD risk. Because diabetes is a public health problem in global, we should pay more attention to the CHD risk of these people; thus, monitoring the progression of CA is necessary.
